# Evolution of the Definition of Rejection in Kidney Transplantation and Its Use as an Endpoint in Clinical Trials

**DOI:** 10.3389/ti.2022.10141

**Published:** 2022-05-20

**Authors:** Jan Ulrich Becker, Daniel Seron, Marion Rabant, Candice Roufosse, Maarten Naesens

**Affiliations:** ^1^ Institute of Pathology, University Hospital Cologne, Cologne, Germany; ^2^ Department of Nephrology and Kidney Transplantation, Vall d’Hebrón University Hospital, Barcelona, Spain; ^3^ Department of Pathology, Hôpital Necker–Enfants Malades, Paris, France; ^4^ Centre for Inflammatory Disease, Department of Immunology and Inflammation, Imperial College London, London, United Kingdom; ^5^ Department of Microbiology, Immunology and Transplantation, KU Leuven, Leuven, Belgium

**Keywords:** biopsy, subclinical rejection, antibody-mediated rejection, T cell-mediated rejection, borderline changes, kidney transplantation outcome

## Abstract

This article outlines the evolving definition of rejection following kidney transplantation. The viewpoints and evidence presented were included in documentation prepared for a Broad Scientific Advice request to the European Medicines Agency (EMA), relating to clinical trial endpoints in kidney transplantation. This request was initiated by the European Society for Organ Transplantation (ESOT) in 2016 and finalized following discussions between the EMA and ESOT in 2020. In ESOT’s opinion, the use of “biopsy-proven acute rejection” as an endpoint for clinical trials in kidney transplantation is no longer accurate, although it is still the approved histopathological endpoint. The spectrum of rejection is now divided into the phenotypes of borderline changes, T cell-mediated rejection, and antibody-mediated rejection, with the latter two phenotypes having further subclassifications. Rejection is also described in relation to graft (dys)function, diagnosed because of protocol (surveillance) or indication (for-cause) biopsies. The ongoing use of outdated terminology has become a potential barrier to clinical research in kidney transplantation. This article presents these perspectives and issues, and provides a foundation on which subsequent articles within this Special Issue of Transplant International build.

## Introduction

The approved histopathological endpoint for clinical trials of kidney transplantation is the presence or absence of biopsy-proven acute rejection (BPAR) (1). This endpoint has not changed for decades, despite many improvements in diagnostic assessment, immunosuppression, and monitoring protocols for kidney transplant recipients, as well as developments in our understanding of the epidemiology and pathophysiology of rejection (2).

Over time, the spectrum of rejection has broadened, with distinctions made between two major subtypes: T cell-mediated rejection (TCMR) and antibody-mediated rejection (AMR) (3). Deeper distinctions have also been made between acute (or active) and chronic phenotypes of TCMR and AMR, as defined in the Banff Classification (2), and subtypes within these phenotypes. In addition, evidence has emerged to indicate that non-specific acute rejection, or early TCMR, is becoming less relevant as the primary endpoint in kidney transplantation (4) because it is no longer considered a strong predictor of graft loss. Ongoing use of outdated terminology and definitions of histopathological endpoints such as BPAR in clinical trials has therefore become a potential barrier to research, particularly for drug development programs that aim specifically at treating only one main rejection subtype.

Furthermore, the strategy of performing protocol biopsies in the early years following transplantation has been adopted by several European centers, to detect subclinical rejection and guide ongoing patient management (5). It has become important, therefore, to consider whether endpoints defined for indication biopsies are also valid for protocol biopsies.

## Rejection Phenotypes

The classification of allograft rejection has often been modified over the years, such that six histological rejection phenotypes are widely described (2, 6):

• Suspicious (borderline) for acute TCMR (henceforth simplified to “borderline changes”)
• Acute TCMR (aTCMR; classified as IA, IB, IIA, IIB, III)
• Chronic active TCMR (caTCMR)
• Acute/active antibody-mediated rejection (aAMR)
• Chronic antibody-mediated rejection (cAMR)
• Chronic active antibody-mediated rejection (caAMR).

Borderline changes represent less severe inflammation scores than aTCMR. The threshold of inflammation used for diagnosis of borderline changes (interstitial inflammation [i]0, <10% of the non-fibrotic cortex; or i1, 10%–25% of the non-fibrotic cortex) varies among centers, because between 2005 and 2017 the Banff Classification stated that retaining the i1 threshold for borderline changes with tubulitis (t) > 0 was permitted (7). However, in 2019 the minimal threshold changed to i1t1, given that several studies indicated that isolated tubulitis in the absence of interstitial inflammation (i0) did not associate with impaired graft outcome—a finding supported by most of those involved in ratifying the Banff 2019 update (7-11). In addition, decreased heterogeneity in center practice is anticipated (11). Banff 2019 also emphasized that “borderline changes” should be known as “borderline (suspicious) for acute TCMR,” to make a clear reference to rejection and treatment (11).

In the 1990s, a diagnosis of aTCMR was based on a clinical definition (i.e., an acute rise of serum creatinine that responded to antirejection therapy) and/or a clinicopathological definition (i.e., acute rejection, being aTCMR or borderline changes in an indication biopsy) (12, 13). The criteria for aTCMR have not changed since the original 1997 Banff Classification and the scores remain based on the presence of interstitial inflammation (i), tubulitis (t), and arteritis (v). However, tubulitis is now considered in all but severely atrophic cortical tubules as either Banff lesion score t or t-IFTA (defined below), whereas previously it was only considered in mildly atrophic or non-atrophic tubules (11).

The impact of inflammation in atrophic areas (i-IFTA) on graft outcomes has been widely demonstrated (8, 14–16), and the effect of i-IFTA on graft survival was not significantly affected by treatment for concomitant aTCMR (15); i-IFTA has also been shown to be related to under-immunosuppression and is more commonly preceded by aTCMR than biopsies without i-IFTA (16, 17), although in some reports the majority of cases with i-IFTA did not have a previous biopsy with rejection (18). These findings suggest that i-IFTA could partly reflect alloimmunity, although further research is warranted. The same applies for tubulitis in moderately atrophic tubules captured as Banff lesion Score t-IFTA (16).

The Banff 2015 meeting noted for the first time that caTCMR could manifest in tubulointerstitial and vascular compartments, and at the 2017 meeting the proposal to include inflammation in areas of fibrosis was incorporated into the consensus classification as caTCMR (2). This classification requires interstitial inflammation involving >25% of the total cortex (ti score 2 or 3) and >25% of the sclerotic cortical parenchyma (i-IFTA score 2 or 3) with moderate tubulitis (t2) involving one or more tubules, not including severely atrophic tubules, while other known causes of i-IFTA are ruled out. Excluding other causes of inflammation in fibrosed areas is important, as i-IFTA is not a specific lesion and can be seen in cases of polyomavirus infection, pyelonephritis, AMR, recurrent glomerulonephritis, and obstruction. Inflammation might instead be an indication of very recent nephron loss as consequence (rather than the cause) of the injury *per se*. The response of caTCMR to increased doses of immunosuppressive therapy has not been studied (2).

In 2001, specific criteria for AMR were introduced (3), linking histopathological changes, presence of C4d, and presence of donor-specific antibodies (DSA). These were revised in 2007 (19) with the introduction of peritubular capillary (PTC) and C4d scores, and cAMR. In 2013, C4d-negative AMR was recognized, and C4d was replaced by a sign of interaction between the DSA and the endothelium (positive C4d or microcirculation inflammation, glomerulitis and peritubular capillaritis [g + PTC] ≥2, or molecular markers) (20). Finally, and importantly, in 2017 the classification for AMR was revised a second time, with acceptance of positive C4d staining as substitute for DSA in the serological criterion for DSA-negative cases and elimination of the suspicious for AMR category (not fulfilling all three criteria). Criteria for AMR were unchanged in 2019.

In addition, rejection phenotypes of kidney transplants are distinguished according to their association with graft (dys)function. Protocol (surveillance) biopsies are performed, per definition, at the time of stable graft function to detect subclinical inflammation (subclinical aTCMR and AMR) (5). Indication (for-cause) biopsies are performed at the time of graft dysfunction.

Finally, although molecular diagnostics of kidney transplant rejection has been validated prospectively in a multicentric fashion (21) and is currently applied for secondary endpoints in clinical trials, we do not consider mRNA expression patterns a valid primary endpoint at this time. Banff has not formally recognized this particular assay and is moving towards an entirely different technological platform (22) which will also need rigorous validation for diagnostic or theranostics use, before being proposed as primary endpoint for clinical trials.

## Conclusions

ESOT has come to the following conclusions:

• The use of BPAR as an endpoint for clinical trials in kidney transplantation is no longer accurate.
  ○ Using outdated and/or non-specific definitions, such as BPAR, compromises the future of high-quality clinical research, especially for interventions that are targeted at one rejection subtype.
• Kidney transplant rejection should be classified by its phenotypes—borderline changes, TCMR, and AMR (the two latter having subtypes), and in relation to the nature of graft (dys)function (i.e., indication [for-cause] vs. protocol [surveillance] biopsies).

### Scientific Advice From the Committee for Medicinal Products for Human Use (CHMP) of the European Medicines Agency (EMA) About These Conclusions

• The CHMP acknowledged that histological subclassifications of rejection have evolved during the last decade.
• The CHMP agreed that the histological subtype of rejection is a useful specification and noted that this detailing might be very informative in profiling efficacy of immunosuppression.
• The CHMP commented that the reason for undertaking a protocol or indication biopsy should be taken into consideration when defining endpoints for clinical trials.
